# Supplementary material for: Randomised controlled trial on the effect of video-conference cognitive behavioural therapy for patients with schizophrenia: a study protocol
Source: BMJ Open. 2023 Sep 11;13(9):e069734. doi: 10.1136/bmjopen-2022-069734 (PMC10496719; doi:10.1136/bmjopen-2022-069734)
Supplement: Author's manuscript [file bmjopen-2022-069734.draft_revisions.pdf]

BMJ Open

BMJ Open is committed to open peer review. As part of this commitment we make the peer review history of every article we publish publicly available.

When an article is published we post the peer reviewers' comments and the authors' responses online. We also post the versions of the paper that were used during peer review. These are the versions that the peer review comments apply to.

The versions of the paper that follow are the versions that were submitted during the peer review process. They are not the versions of record or the final published versions. They should not be cited or distributed as the published version of this manuscript.

BMJ Open is an open access journal and the full, final, typeset and author-corrected version of record of the manuscript is available on our site with no access controls, subscription charges or pay-per-view fees (http://bmjopen.bmj.com).

If you have any questions on BMJ Open's open peer review process please email info.bmjopen@bmj.com

BMJ Open

Randomised controlled trial on the effect of video-conference cognitive behavioural therapy for patients with schizophrenia: A study protocol

Journal:	*BMJ Open*
Manuscript ID	bmjopen-2022-069734
Article Type:	Protocol
Date Submitted by the Author:	31-Oct-2022

Complete List of Authors:	Katsushima, Masayuki; Chiba University Graduate School of Medicine School of Medicine, Departments of Cognitive Behavioral Physiology; Teikyo Heisei University - Chiba Campus, Department of Rehabilitation, Faculty of Health Care and Medical Sports<br>Nakamura, Hideki; Chiba University Graduate School of Medicine School of Medicine, Departments of Cognitive Behavioral Physiology; Jikei University School of Medicine, Department of Nursing, Faculty of Medicine<br>Hanaoka, Hideki; Chiba University Hospital, Clinical Research Center<br>Shiko, Yuki; Chiba University Hospital, Clinical Research Center; Chiba University,<br>Komatsu, Hideki; Kameda Medical Center, Department of Psychiatry and Psychosomatic Medicine<br>Shimizu, Eiji; Chiba University, Department of Cognitive Behavioral Physiology, Graduate School of Medicine; Chiba University, Research Center for Child Mental Development, Graduate School of Medicine

Keywords:	Schizophrenia & psychotic disorders < PSYCHIATRY, MENTAL HEALTH, Telemedicine < BIOTECHNOLOGY & BIOINFORMATICS

SCHOLARONE™
Manuscripts

Title page

Title of this article

Randomised controlled trial on the effect of video-conference cognitive behavioural therapy for patients with schizophrenia: A study protocol

Author name

Masayuki Katsushima[1,2] , Hideki Nakamura[1,3] , Hideki Hanaoka[4] , Yuki Shiko[4] , Hideki Komatsu[5] , Eiji Shimizu[1,6,7]

Author affiliations

[1] Departments of Cognitive Behavioral Physiology, Chiba University Graduate School of Medicine, Chiba, Japan

[2] Department of Rehabilitation, Faculty of Health Care and Medical Sports, Teikyo Heisei University, Chiba, Japan

[3] Department of Nursing, Faculty of Medicine, The Jikei University School of Medicine, Tokyo, Japan

[4] Clinical Research Center, Chiba University Hospital, Chiba, Japan

[5] Department of Psychiatry and Psychosomatic Medicine, Kameda Medical Center, Chiba, Japan

[6] Cognitive Behavioral Therapy Center, Chiba University Hospital, Chiba, Japan

[7] Research Center for Child Mental Development, Chiba University, Chiba, Japan

Contact address of corresponding author

Masayuki Katsushima[1,2]

[1] 1-8-1 Inohana, Chuo-ku, Chiba-shi, Chiba, 260-8670 Japan +81-43-226-2027, axaa4738@chiba-u.jp

Graduate school doctoral course

Co-author : Email address

Hideki Nakamura：hnakamura@jikei.ac.jp

Hideki Hanaoka : hanaoka.hideki@mac.com

Yuki Shiko : shiko_yuki@chiba-u.jp

Hideki Komatsu : chibakomatsu@gmail.com

Eiji Shimizu : eiji@faculty.chiba-u.jp

Contributors:

MK and ES designed the study and developed the protocol with HH and YS; MK, ES, and HK recruited the patients; MK, HN, and ES managed the preparation of the manuscript; MK wrote the first draft of the manuscript; MK, HN, and ES wrote the manuscript. All the authors contributed to and approved the final version of the manuscript.

Funding:

This work was supported by a Grant-in-Aid for Scientific Research (Grant No. 21K11197, Japan Society for the Promotion of Science).

For peer review only

Competing interests:

None declared.

Patient consent

Obtained

Ethics approval

The study protocol has been approved by the Institutional Review Board of the Chiba University Hospital (reference number: G2020031) on January 2021. The clinical trial registration number is UMIN000043396.

English proofreading

We would like to thank Editage (www.editage.com) for English language editing.

Total word count of the article

2484 words.

Number of figures and tables

1 figure.

keyword

①Schizophrenia ②Cognitive Behavioural Therapy ③Remote ④Accessibility

For peer review only

ABSTRACT

Introduction Cognitive behavioural therapy for psychosis (CBTp) has proven effective. However, compared to cognitive behavioural therapy for depression and anxiety, the spread of CBTp in clinical practice is minimal. The present study designed a randomised controlled trial research protocol to evaluate whether real-time remote video-conference CBTp (vCBTp) could facilitate access to psychosocial interventions and improve symptoms effectively for patients with schizophrenia.

Methods and analysis This exploratory RCT will consist of two parallel groups (vCBTp+UC and UC alone) of 12 participants (n=24) diagnosed with schizophrenia, schizoaffective disorder, or paranoid disorder, who remain symptomatic following pharmacotherapy. Seven 50-minute weekly vCBTp interventions will be administered to test efficacy. The primary outcome will be the positive and negative syndrome scale (PANSS) score at week 8. The secondary outcome will be the Beck Cognitive Insight Scale to assess insight, the Patient Health Questionnaire-9 (PHQ-9) to assess depression, the Generalized Anxiety Disorder-7 (GAD-7) to assess anxiety, the EuroQol five-dimensional questionnaire (EQ-5D) to assess quality of life, and the impact of Event Scale-Revised (IES-R) to assess subjective distress about a specific stressful life event. We will take all measurements at 0 weeks (baseline) and at eight weeks (post-intervention), and apply intention-to-treat analysis.

Ethics and dissemination We will conduct this study in the outpatient department of Cognitive Behavioral Therapy Center at Chiba University Hospital. Further, all participants will be informed of the study and will be asked to sign consent forms. We will report according to the Consolidated Standards of Reporting Trials (CONSORT).

Trial registration number The clinical trial registration number is UMIN000043396.

Strengths and limitations of this study

► To the best of our knowledge, this study will be the first randomised controlled trial focusing on individualized cognitive behavioural therapy using a real-time video-conference system as an additional treatment for patients diagnosed schizophrenia with persistent symptoms after antipsychotic medication.

For peer review only

►This study will suggest the effectiveness of remote psychosocial support for patients with schizophrenia, and the results will significantly contribute to developing future treatment systems.

►This study has limitations. It is a single-center clinical study. The sample size must be small. The follow-up study is set up as a separate study.

For peer review only

INTRODUCTION

Based on the fifth edition of the Diagnostic and Statistical Manual of Mental Disorders (DSM-5), schizophrenia is a mental disorder associated with functional disability, poor quality of life, and early death.[1] Schizophrenia is a severe, lifelong mental disorder that affects approximately 1% of the world's population.[2] The illness tends to develop between the ages of 16 and 30 years.[3] Individuals with schizophrenia use a substantial amount of healthcare services. This condition imposes a significant economic burden on both the patients and their families, and on society as a whole [4]

Standard treatment for schizophrenia

The treatment of schizophrenia is multi-dimensional and comprehensive. The first treatment option is pharmacotherapy with antipsychotic medications and psychological interventions, such as cognitive-behavioral therapy, as recommended by the National Institute of Health and Clinical Excellence.[5] The American Psychiatric Association guidelines,[6] and the Schizophrenia Patient Outcomes Research Team,[7] recommend psychosocial interventions in combination with pharmacotherapy. Comprehensive treatment measures include individual and group psychotherapy, psychoeducation, occupational therapy, family therapy, and psychosocial interventions such as social skills training.[8]

Cognitive behavioural therapy for schizophrenia

Cognitive behavioural therapy for psychosis (CBTp) has been shown to reduce positive symptoms and improve coping skills in Europe and the United States.[9] Kuller *et al*[10] reported that CBTp is used in 58% of U.S. medical facilities and 91.3% of U.K. medical facilities. Wykes *et al*[11] reported an effect size of 0.40 (0.25-0.55) in a meta-analysis of 33 RCTs (n=1,964) on CBTp. Jauhar *et al*[12] found an effect size of 0.33 for general symptoms in a meta-analysis of 34 studies on CBTp for schizophrenia. The National Institute of Health and Clinical Excellence recommends a session structure of 16 sessions or more.[5] However, despite the evidence, the number of practitioners is insufficient; thus, it is challenging for patients to access CBTp.[13] Additionally, CBTp is important because patients' characteristics and problems are diverse due to the illness's nature. It is essential to be flexible in dealing with mood and thoughts, realistic and rational thoughts, and delusional and pathological experiences.[14]

There are reports of an association between intrusive thoughts of post-traumatic experiences and schizophrenia.[15,16] There is also a discussion of the potential for CBTp to address intrusive thoughts.[17]

In the future, it will be desirable to establish an effective implementation system for CBTp and

For peer review only - http://bmjopen.bmj.com/site/about/guidelines.xhtml

increase the number of practitioners in clinical practice.

Evidence of low-intensity CBTp has been collected overseas, and its effectiveness has been recognized,too.[18,19] Turkington *et al*[20,21] found that community psychiatric nurses' provision of low-intensity CBTp, comprising six sessions, effectively increased awareness of the illness. Overall, six sessions of a six-week low-intensity CBTp provided by community psychiatric nurses improved the understanding of illness, general status, and depression. This suggests that CBTp offered by health care professionals may also be effective. Recently, studies have begun investigating CBTp that can be effectively administered online using the Internet.[22-25]

Video-conference cognitive behavioural therapy for psychosis

The number of CBTp practitioners is limited, and their use in clinical practice is much less frequent. As a remedy to this problem, there is growing interest in using video-conferenced systems to provide CBT, known as vCBT.[26] This approach has the advantage of providing access to CBT for patients living in remote areas and enabling remote treatment through interactive, real-time communication between the therapist and patient.[27] Although therapist guided Internet-based CBT is shown to be not inferior to face-to-face CBT in treating anxiety, depression, insomnia and somatic disorders,[28] there is less reports about Internet-based CBTp. Up to this point in 2022, to the best of our knowledge, no studies have examined the efficacy of individual CBTp using a video-conference system (vCBTp) for schizophrenia.[29]

Objective

This article describes the study protocol of a randomised controlled trial designed to evaluate the clinical efficacy of seven sessions of vCBTp adjuncts to usual care (UC) compared to UC alone among patients with schizophrenia whose symptoms do not remit after pharmacotherapy.

METHODS AND ANALYSIS

Study design

The study is designed as a single center, assessor-blind, two-arm, parallel, prospective, randomised, controlled trial comprising a 7-week treatment regimen. Participants will be allocated to vCBTp plus UC or UC alone (Figure 1). We will report according to the Consolidated Standards of Reporting Trials (CONSORT) and the SPIRIT reporting guidelines.[30]

Participants

Inclusion criteria of participants in this study are between 16 and 65 years of age, primary diagnosis of schizophrenia, schizoaffective disorder, or delusional disorder according to the fifth edition of the

For peer review only - http://bmjopen.bmj.com/site/about/guidelines.xhtml

Diagnostic and Statistical Manual of Mental Disorders (DSM-5), and who are found competent to consent to the study in the MacArthur Competence Assessment Tool for Clinical Research (MacCAT-CR),[31,32] having at least three out of seven points on any one of the seven positive and negative syndrome scale (PANSS) positive symptoms. In addition, participants will be required to have Internet and computer access and an environment that allowed them to take the vCBTp. The exclusion criteria are severe addiction and substance dependence, such as alcohol or drug dependence, mental retardation, neurocognitive disorders, or predicted risk of self-harm or other harm due to worsening symptoms, reported by the participants, their family members, or their psychiatrists.

Two researchers (a psychiatrist [ES] and a researcher [MK]) will evaluate and confirm the patients' eligibility including diagnosis, treatment history by their psychiatrists, the suitability of the symptoms and their ability to consent to participate in the study.

Details of recruitment

The researchers will recruit participants through websites, posters and leaflets placed at medical institutions in Chiba Prefecture, Japan, from April 2021 to March 2023 until 24 participants will be enrolled. Participants will be required to obtain permission from their psychiatrists before enrollment in the study, and continue to receive medical care including pharamcotherapy from their psychiatrists. The study will be conducted in the Cognitive Behavioural Therapy Center outpatient clinic at Chiba University Hospital.

Intervention methods

Participants will not be restricted to changes in their medications by their psychiatrists during the study period. If clinically appropriate, they will be allowed to participate in usual psychosocial intervention programs, such as social skills training at psychiatric daycare or nightcare.

However, to properly evaluate the effectiveness of vCBTp, participants may not receive other cognitive behavioural therapis or specific psychotherapies including psychoanalytic therapy, Morita therapy, insight therapy, hypnotherapy, brain stimulation therapies including electroconvulsive therapy or magnetic stimulation therapies. The vCBTp+UC group will receive the text material developed by the research team by mail. All seven sessions will be conducted according to the text material. The therapist will assign homework to the participants at the end of each session, as described in the text material.

For peer review only - http://bmjopen.bmj.com/site/about/guidelines.xhtml

The vCBTp program for schizophrenia was developed by two researchers (ES and MK). The treatment lasts over seven weeks (once a week, 50 minutes per session). It included elements incorporated into CBT for depression and anxiety, in general. This protocol primarily focuses on cognitive restructuring. The components are as follows: (1) Assessment and goal setting, (2) externalization of stress in present life, (3) cognitive restructuring of stress in present life, (4) mood change and relaxation, (5) externalization of past stressful experiences, (6) reappraisal of past stressful experiences, and (7) relapse prevention. As described in the introdction, we focus on subjective distress from intrusive thoughts of stressful experiences in patients with schizophrenia.[15,16,17]

Each session includes examples of cognitive restructuring for patients with schizophrenia and homework assignments. The therapist conducting the session and the participant will be connected remotely in real-time via a web conferencing system between the participant's house and Chiba University Hospital for cognitive-behavioural therapy. Participants will have to work on the vCBTp and receive supportive feedback on the requested homework. Participants will also be allowed to email the research office (MK) if they have questions regarding the contents.

Outcomes

Baseline and clinical characteristics

The baseline and clinical characteristics included sex, age, marital status, employment status, age at onset of schizophrenia, duration of illness and treatment history including the name of the antipsychotic to which the patient has developed resistance, current drug titers at baseline, and any changes in conventional treatment during the study period will be collected by researchers.

Primary outcome

The primary outcome is change in the PANSS[33] total score at week 8 from baseline, The PANSS is a 30-item interview-based assessment consisting of seven positive symptoms, seven negatives, and 16 general psychopathology items. Each subscale is scored on a scale of 1-7 points. The PANSS is an objective scale for assessing schizophrenia symptoms in clinical and experimental studies and has become a worldwide standard for reliability and validity. An assessor (a researcher, HN) blind to treatment allication will be evaluated the PANSS by web conferencing system at baseline and at week 8, respectively.

Secondary outcomes

The secondary outcomes are the following;

We will evaluate the sum of positive symptoms, negative symptoms, and general psychopathology

For peer review only - http://bmjopen.bmj.com/site/about/guidelines.xhtml

extracted from the PANSS sucscale score, resceptively. The total score of the seven positive symptoms subscale on the PANSS ranges from 7 (no symptoms) to 49 (severe positive symptoms). The total score of the seven negative symptoms subscale ranges from 7 (no symptoms) to 49 (severe negative symptoms). The total score of the 16 items of general psychopathology subscale ranged from 16 (no symptoms) to 112 (severity of psychopathology).

The Japanese version of the Beck Cognitive Insight Scale (BCIS-J)[34] will be used to assess cognitive insight. A total of 15 items of the BCIS-J are divided into nine items of self-reflection (0-36) and six items of self-confidence (0-24). The lower the subtotal of nine self-reflection items minus six self-confidence items, the lower the cognitive pathology.

The Impact of Event Scale-Revised(IES-R)[35] will be used to assess subjective distress for a specific stressful life event. The 22 items (0-88) of IES-R comprise eight intrusive symptoms, eight avoidance symptoms, and six hyperarousal symptoms, and can measure symptoms in persons exposed to traumatic experiences.

The Patient Health Questionnaire-9 (PHQ-9)[36] will be used to assess depressive symptoms. PHQ-9 comprise nine-item ranges from 0 (no depressive symptoms) to 27 (severe depressive symptoms).

The Generalized Anxiety Disorder-7(GAD-7)[37] will be used to assess anxiety. GAD-7 comprise seven-item ranged from 0 (no anxiety symptoms) to 21 (severe anxiety symptoms).

The EuroQol five-dimensional questionnaire (EQ-5D-5L)[38] will be used to assess QOL. It comprises five items and assesses the quality of life on a five-point Likert scale from 1 (not severe) to 5 (severe). It is the most commonly used measure of quality-adjusted life-years worldwide.

In addition, we will evaluate the chlorpromazine equivalent to daily prescriptions for antipsychotic medications.[39]

Therapists will ask the participants about their experience of adverse events during each assessment. All measures will be assessed at week 0 (baseline) and week 8 (post-intervention), and the results will be analyzed per the intention-to-treat principle.

Sample size

Primarily, this study aims to test the superiority of video confenrence cognitive-behavioral therapy (vCBT) on symptom improvement in a group of patients with schizophrenia compared to a usual care (UC) group. We refer to a previous study by Morrison *et al*[40] in which CBT and antipsychotic groups were compared. Assuming that the mean difference between the vCBT plus US group and UC alone group after the intervention is 12 points and the standard deviation of the pooled UC group is eight, the number of patients in each group was calculated as 11 for a significance level of 5% on

For peer review only - http://bmjopen.bmj.com/site/about/guidelines.xhtml

both sides and a power of 90%. Therefore, the target number of patients was set at 12 in each group, assuming a dropout rate of 10% for 24 patients.

Randomisation and assessor-blindness

After baseline assessment, participants will be randomly assigned to the UC group or vCBTp+UC in a 1:1 ratio, using the minimization method to ensure a balance between baseline PANSS total score (PANSS≥51) and sex. The PANSS values are based on a study by Naeem *et al*[41] Each participant will be randomly assigned to one of the two treatments. The assessor (a researcher, HN) will be not informed of the participant's allocation group throughout the study period for assessor-blindness.

Data analysis plan

Statistical analysis and reporting of the study will be conducted per the CONSORT guidelines. The primary analysis will be based on the intention-to-treat principle. For the primary outcome, the changes in the PANSS total score at week 8 from will be calculated for both groups at a two-sided significance level of 5% and a two-sided 95% confidence interval. The treatment responses in each group will be statistically analyzed and compared. Secondary outcomes will be analyzed to provide additional insights into the primary endpoints. The secondary outcomes will not be adjusted for multiplicity.

Ethics and dissemination

The study protocol has been approved by the Institutional Review Board of the Chiba University Hospital (reference number: G2020031) on January 2021. The clinical trial registration number is UMIN000043396.

We will conduct this study at the Outpatient Department of the Cognitive Behavioural Therapy Center at Chiba University Hospital. Prospective participants will be informed of the purpose of the study and asked about their willingness to participate when they contact the researchers. Participation is voluntary and complete anonymity will be guaranteed. Each participant will be asked to provide written informed consent to participate in the study after MacCAT-CR confirms that the participant is competent to consent to the clinical trial. All participants will receive UC from their psychiatrists, and half of the participants will receive vCBTp in addition to UC. Participants randomised to the UC arm will be eligible to receive vCBTp in an ancillary study (UMIN000044244) after completion of the study. A blinded assessor will evaluate the participants at each assessment point (Weeks 0 and

For peer review only

For peer review only - http://bmjopen.bmj.com/site/about/guidelines.xhtml

8). All adverse events will be reported, and serious ones will be immediately reported to the Clinical Trial Review Committee of Chiba University Hospital. Moreover, they will be registered in the hospital’s risk management system. An independent data monitoring committee then properly review detailed records of the progress of the clinical trial, key efficacy variables, and safety data and recommend the trial’s continuation, modification, or termination to the clinical investigators accordingly. Regardless of the outcome, the trial results will be published in an international journal. This study will be conducted and reported per Consolidated Standards of Reporting Trials (CONSORT) recommendations.

DISCUSSION

This study will address the effect of vCBTp for treatment-resistant schizophrenia.[13,20,21,42,43] The findings of this study will provide valuable evidence to facilitate the development of new therapeutic support modalities and promote increased treatment options for patients. The anticipated limitations of this study are as follows. First, because this study is a single-center study, it has less generalizability than those conducted in multi-center studies. Second, the sample size must be small. Third, the follow-up study is set up as a separate study. Therefore, verifying the retention of this effect in this study is not easy.The present study is a pilot study, and the study design is based on consideration of patient burden. If this study is successfully completed and proven safe and a certain level of efficacy is suggested, a full-scale study with a larger sample size should be planned.

For peer review only

For peer review only - http://bmjopen.bmj.com/site/about/guidelines.xhtml

REFERENCES

1. American Psychiatric Association. Diagnostic and statistical manual of mental disorders. 5th ed. Washington: APA, 2013.
2. Saha S, Chant D, Welham J, et al. A systematic review of the prevalence of schizophrenia. ***PLoS Med*** 2005:;2:e141.
3. Mueser KT, McGurk SR. Schizophrenia. ***Lancet*** 2004; 9426:2063-72.
4. G. ková, T. Almá, A. Millier, et al. Direct healthcare cost of schizophrenia – European overview. **Eur Psychiatry** 2018; 48:79-92.
5. National institute for Health and Care Excellence. Psychosis and schizophrenia in adults: prevention and management. NICE, 2014.
6. Keepers GA, Fochtmann LJ, Anzia JM, et al. The American Psychiatric Association Practice Guideline for the Treatment of Patients with Schizophrenia. ***Am Psychiatr Publ*** 2020; 4:493-497.
7. Dixon LB, Dickerson F, Bellack AS, et al. The Schizophrenia Patient Outcomes Research team (PORT): The 2009 schizophrenia PORT psychosocial treatment recommendations and summary statements. ***Schizophr Bull*** 2010; 36:48-70.
8. Bighelli I, Rodolico A, Mieres HG, et al. Psychosocial and psychological interventions for relapse prevention in schizophrenia: a systematic review and network meta-analysis. ***Lancet Psychiatry*** 2021; 11:969-980.
9. Mankiewicz PD, Turner C. Cognitive Restructuring and Graded Behavioural Exposure for Delusional Appraisals of Auditory Hallucinations and Comorbid Anxiety in Paranoid Schizophrenia. ***Case Rep Psychiatry*** 2014; 2014:124564.
10. Kuller AM, Ott BD, Goisman RM, et al. Cognitive behavioral therapy and schizophrenia. A survey of clinical practices and views on efficacy in the United States and United Kingdom. ***Community Ment Health J*** 2010; 46:2-9.
11. Wykes T, Steel C, Everitt B, et al. Cognitive behavior therapy for schizophrenia: effect sizes, clinical models and methodological rigor. ***Schizophr Bull*** 2008; 34:523-537.
12. S Jauhar, P J McKenna, Radua J, Fung E, Salvador R, Laws K.R. Cognitive-behavioural therapy for the symptoms of schizophrenia: systematic review and meta-analysis with examination of potential bias. ***Br J Psychiatry*** 2014; 204:20-9.
13. Shafran R, Clark DM, Fairburn CG, et al. Mind the gap: Improving the dissemination of CBT. ***Behav Res Ther*** 2009; 47:902-9.
14. Ishigaki T, Kikuchi A, Matsumoto K, et al. Cognitive behavior Therapy for psychosis (CBTp) Learning through Case Studies. Kongo Publishing Co. Tokyo,2019.
15. Malcolm CP, Picchioni MM, Ellett L, et al. Intrusive prospective imagery, posttraumatic intrusions and anxiety in schizophrenia. ***Psychiatry Res*** 2015;230: 899-904.

For peer review only

16. Morrison AP, Baker CA. Intrusive thoughts and auditory hallucinations: a comparative study of intrusions in psychosis. ***Behav Res Ther*** 2000;38:1097-1106

17. O'Driscoll C, Mason O, Brady F, et al. Process analysis of trauma-focused cognitive behavioural therapy for individuals with schizophrenia.***Psychol Psychother*** 2016;89:117-32

18. Hazell CM, Hayward M, Cavanagh K, et al. A systematic review and meta-analysis of low intensity CBT for psychosis. ***Clin Psychol Rev*** 2016; 45:183-192.

19. Malik N, Kingdon D, Pelton J, et al. Effectiveness of brief cognitive-behavioral therapy for schizophrenia delivered by mental health nurses: relapse and recovery at 24 months. ***Clin Psychiatry*** 2009; 70:201-7.

20. Turkington D, Kingdon D, Turner T, et al. Effectiveness of a brief cognitive-behavioral therapy intervention in the treatment of schizophrenia. ***Br J of Psychiatry*** 2002; 180:523-7.

21. Brabban A, Rai S, Turkington D. Predictors of Outcome in Brief Cognitive Behavior Therapy for Schizophrenia. ***Schizophr Bull*** 2009; 35: 859-864.

22. Gottlieb JD, Romeo KH, Penn DL, et al. Web-based cognitive-behavioral therapy for auditory hallucinations in persons with psychosis: A pilot study. ***Schizophr Res*** 2013; 145:82-7.

23. Gottlieb JD, Gidugu V, Maru, M, et al. Randomized controlled trial of an internet cognitive behavioral skills-based program for auditory hallucinations in persons with psychosis. ***Psychiatr Rehabil J*** 2017;40: 283-292.

24. Rüegg N, Moritz S, Berger T, et al. An internet-based intervention for people with psychosis (EviBaS): study protocol for a randomized controlled trial. ***BMC Psychiatry*** 2018; 18:102.

25. Westermann S, Rüegg N, Lüdtke T, et al. Internet-based self-help for psychosis: Findings from a randomized controlled trial. ***J Consult Clin Psychol*** 2020;88:937-950.

26. Matsumoto K, Sutoh C, Asano K, et al. Internet-Based Cognitive Behavioral Therapy with Real-Time Therapist Support via Videoconference for Patients with Obsessive-Compulsive Disorder, Panic Disorder, and Social Anxiety Disorder: Pilot Single-Arm Trial. ***J Med Internet Res*** 2018;20: e12091.

27. Lecomte T, Amal AB, Francoeur A, et al. Group therapy via videoconferencing for individuals with early psychosis: A pilot study. ***Early Interv Psychiatry*** 2021; 15:1595-1601.

28. Carlbring P, Andersson G, Cuijpers P, et al. Internet-based vs. face-to-face cognitive behavior therapy for psychiatric and somatic disorders: an updated systematic review and meta-analysis. ***Cogn Behav Ther*** 2018; 47:1-18.

29. Kopelovich SL, Turkington D. Remote CBT for Psychosis During the COVID-19 Pandemic: Challenges and Opportunities. ***Community Ment Health J*** 2021.;57:30–34.

30. Chan A-W, Tetzlaff JM, Gøtzsche PC, et al. SPIRIT 2013 Explanation and Elaboration: Guidance for protocols of clinical trials. ***BMJ***. 2013;346:e7586

31. Appelbaum P, Grisso T. The MacArthur Competence Assessment Tool for Clinical Research

For peer review only

(MacCAT-CR). Professional Resource Press: Sarasota, FL,2001.

32. Fovet T, Amad A, Thomas P, et al. Schizophrenia and informed consent to research. Encephale 2015; 41:394-402

33. Kay SR, Fiszbein A, Opler LA. The Positive and Negative Syndrome Scale (PANSS) for Schizophrenia. ***Schizophr Bull*** 1987; 13:261-276.

34. Uchida T, Matsumoto K, Kikuchi K, et al. atsuoka. Psychometric properties of the Japanese version of the Beck Cognitive Insight Scale: relation of cognitive insight to clinical insight. ***Psychiatry Clin Neurosci*** 2009; 63:291-7.

35. Asukai N, Kato H, kawamura N, et al. Reliability and validity of the Japanese-language version of the impact of event scale-revised (IES-R-J): Four studies of different traumatic events. ***J Nerv Ment Dis*** 2002; 190:175-182.

36. Muramatsu K, Miyaoka H, Kamijima K, et al. The patient health questionnaire, Japanese version: validity according to the mini-international neuropsychiatric interview-plus. ***Psychol Rep*** 2007; 101:952-60.

37. Spitzer RL, Kroenke K, Williams JB, et al: A brief measure for assessing generalized anxiety disorder: the GAD-7. ***Arch Intern Med*** 2006; 166:1092-7.

38. Feng YS, Kohlmann T, Janssen MF, et al. Psychometric properties of the EQ-5D-5L: a systematic review of the literature. ***Qual Life Res*** 2021; 30:647-673.

39. Woods SW. Chlorpromazine equivalent doses for the newer atypical antipsychotics. ***J Clin Psychiatry*** 2003; 64:663-7.

40. Morrison AP, Law H, Carter L, et al. Antipsychotic drugs versus cognitive behavioural therapy versus a combination of both in people with psychosis: a randomized controlled pilot and feasibility study. ***Lancet Psychiatry*** 2018; 5: 411-423.

41. Naeem F, Johal R, McKenna C, et al. Cognitive Behavior Therapy for psychosis based Guided Self-help (CBTp-GSH) delivered by frontline mental health professionals: Results of a feasibility study. ***Schizophr Res*** 2016; 173:69-74.

42. Pillny M, Lincoln TM. Modern cognitive behavior therapy of psychotic disorders. ***Nervenarzt*** 2020; 91:43-49.

43. Jolley S, Onwumere J, Kuipers E, et al. Increasing access to psychological therapies for people with psychosis: predictors of successful training. ***Behav Res Ther*** 2012; 50:457-62.

For peer review only

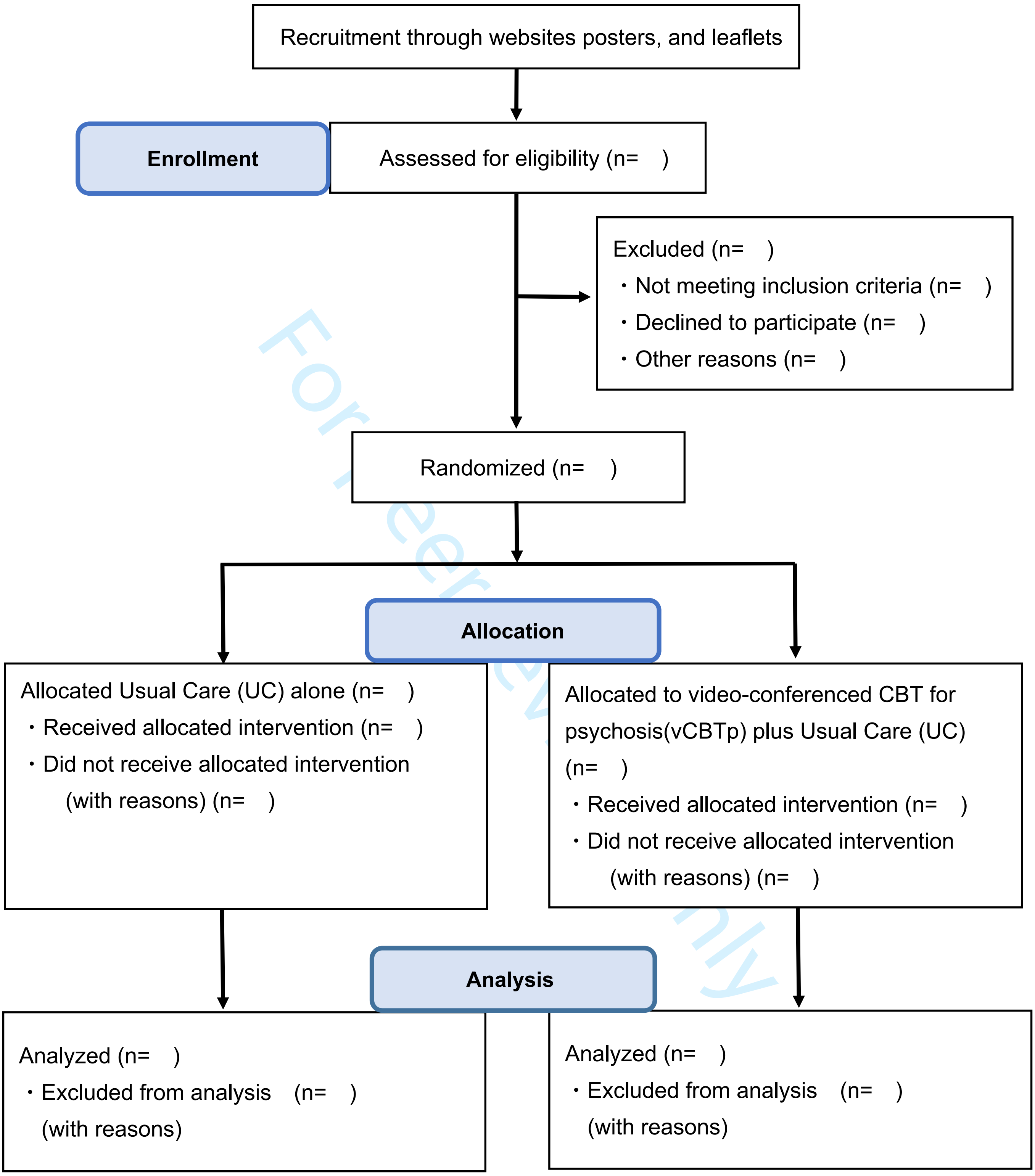

Figure1. A CONSORT flow diagram of the study

For peer review only

Reporting checklist for protocol of a clinical trial.

Based on the SPIRIT guidelines.

Instructions to authors

Complete this checklist by entering the page numbers from your manuscript where readers will find each of the items listed below.

Your article may not currently address all the items on the checklist. Please modify your text to include the missing information. If you are certain that an item does not apply, please write "n/a" and provide a short explanation.

Upload your completed checklist as an extra file when you submit to a journal.

In your methods section, say that you used the SPIRITreporting guidelines, and cite them as:

Chan A-W, Tetzlaff JM, Gøtzsche PC, Altman DG, Mann H, Berlin J, Dickersin K, Hróbjartsson A, Schulz KF, Parulekar WR, Krleža-Jerić K, Laupacis A, Moher D. SPIRIT 2013 Explanation and Elaboration: Guidance for protocols of clinical trials. BMJ. 2013;346:e7586

		Reporting Item	Page Number
**Administrative information**			
Title	#1	Descriptive title identifying the study design, population, interventions, and, if applicable, trial acronym	Listed on the title page.

For peer review only

Trial registration	#2a	Trial identifier and registry name. If not yet registered, name of intended registry	Page 6 of the manuscript.
Trial registration: data set	#2b	All items from the World Health Organization Trial Registration Data Set	Page 6 of the manuscript.
Protocol version	#3	Date and version identifier	n/a
Funding	#4	Sources and types of financial, material, and other support	Listed on the title page.
Roles and responsibilities: contributorship	#5a	Names, affiliations, and roles of protocol contributors	Listed on the title page.
Roles and responsibilities: sponsor contact information	#5b	Name and contact information for the trial sponsor	n/a<br>Competing interests are none.
Roles and responsibilities: sponsor and funder	#5c	Role of study sponsor and funders, if any, in study design; collection, management, analysis, and interpretation of data; writing of the report; and the decision to submit the report for publication, including whether they will have ultimate authority over any of these activities	n/a<br>Competing interests are none.
Roles and responsibilities: committees	#5d	Composition, roles, and responsibilities of the coordinating centre, steering committee, endpoint adjudication committee, data management team, and	Page 6 of the manuscript.

For peer review only

		other individuals or groups overseeing the trial, if applicable (see Item 21a for data monitoring committee)	
**Introduction**			
Background and rationale	#6a	Description of research question and justification for undertaking the trial, including summary of relevant studies (published and unpublished) examining benefits and harms for each intervention	Page 1 of the manuscript.
Background and rationale: choice of comparators	#6b	Explanation for choice of comparators	Page 2 of the manuscript.
Objectives	#7	Specific objectives or hypotheses	Page 2 of the manuscript.
Trial design	#8	Description of trial design including type of trial (eg, parallel group, crossover, factorial, single group), allocation ratio, and framework (eg, superiority, equivalence, non-inferiority, exploratory)	Page 2 of the manuscript.
**Methods: Participants, interventions, and outcomes**			
Study setting	#9	Description of study settings (eg, community clinic, academic hospital) and list of countries where data	Page 3 of the manuscript.

For peer review only

		will be collected. Reference to where list of study sites can be obtained	
Eligibility criteria	#10	Inclusion and exclusion criteria for participants. If applicable, eligibility criteria for study centres and individuals who will perform the interventions (eg, surgeons, psychotherapists)	Page 3 of the manuscript.
Interventions: description	#11a	Interventions for each group with sufficient detail to allow replication, including how and when they will be administered	Page 3 of the manuscript.
Interventions: modifications	#11b	Criteria for discontinuing or modifying allocated interventions for a given trial participant (eg, drug dose change in response to harms, participant request, or improving / worsening disease)	Page 4 of the manuscript.
Interventions: adherance	#11c	Strategies to improve adherence to intervention protocols, and any procedures for monitoring adherence (eg, drug tablet return; laboratory tests)	Page 4 of the manuscript.
Interventions: concomitant care	#11d	Relevant concomitant care and interventions that are permitted or prohibited during the trial	Page 4 of the manuscript.
Outcomes	#12	Primary, secondary, and other outcomes, including the specific measurement variable (eg, systolic blood pressure), analysis metric (eg, change from baseline, final value, time to event), method of aggregation (eg, median, proportion), and time point for each outcome. Explanation of the clinical relevance of	Page 4-5 of the manuscript.

For peer review only

		chosen efficacy and harm outcomes is strongly recommended	
Participant timeline	#13	Time schedule of enrolment, interventions (including any run-ins and washouts), assessments, and visits for participants. A schematic diagram is highly recommended (see Figure)	Page 3 of the manuscript.
Sample size	#14	Estimated number of participants needed to achieve study objectives and how it was determined, including clinical and statistical assumptions supporting any sample size calculations	Page 5 of the manuscript.
Recruitment	#15	Strategies for achieving adequate participant enrolment to reach target sample size	Page 3 of the manuscript.
**Methods: Assignment of interventions (for controlled trials)**			
Allocation: sequence generation	#16a	Method of generating the allocation sequence (eg, computer-generated random numbers), and list of any factors for stratification. To reduce predictability of a random sequence, details of any planned restriction (eg, blocking) should be provided in a separate document that is unavailable to those who enrol participants or assign interventions	Page 5 of the manuscript.

For peer review only

Allocation concealment mechanism	#16b	Mechanism of implementing the allocation sequence (eg, central telephone; sequentially numbered, opaque, sealed envelopes), describing any steps to conceal the sequence until interventions are assigned	Page 5 of the manuscript.
Allocation: implementation	#16c	Who will generate the allocation sequence, who will enrol participants, and who will assign participants to interventions	Page 5 of the manuscript.
Blinding (masking)	#17a	Who will be blinded after assignment to interventions (eg, trial participants, care providers, outcome assessors, data analysts), and how	Page 5 of the manuscript.
Blinding (masking): emergency unblinding	#17b	If blinded, circumstances under which unblinding is permissible, and procedure for revealing a participant's allocated intervention during the trial	Page 6 of the manuscript.
**Methods: Data collection, management, and analysis**			
Data collection plan	#18a	Plans for assessment and collection of outcome, baseline, and other trial data, including any related processes to promote data quality (eg, duplicate measurements, training of assessors) and a description of study instruments (eg, questionnaires, laboratory tests) along with their reliability and	Page 4-5 of the manuscript.

For peer review only

		validity, if known. Reference to where data collection forms can be found, if not in the protocol	
Data collection plan: retention	#18b	Plans to promote participant retention and complete follow-up, including list of any outcome data to be collected for participants who discontinue or deviate from intervention protocols	Page 5 of the manuscript.
Data management	#19	Plans for data entry, coding, security, and storage, including any related processes to promote data quality (eg, double data entry; range checks for data values). Reference to where details of data management procedures can be found, if not in the protocol	Page 6 of the manuscript.
Statistics: outcomes	#20a	Statistical methods for analysing primary and secondary outcomes. Reference to where other details of the statistical analysis plan can be found, if not in the protocol	Page 6 of the manuscript.
Statistics: additional analyses	#20b	Methods for any additional analyses (eg, subgroup and adjusted analyses)	Page 6 of the manuscript.
Statistics: analysis population and missing data	#20c	Definition of analysis population relating to protocol non-adherence (eg, as randomised analysis), and any statistical methods to handle missing data (eg, multiple imputation)	Page 6 of the manuscript.

For peer review only

Methods: Monitoring

Data monitoring: formal committee	#21a	Composition of data monitoring committee (DMC); summary of its role and reporting structure; statement of whether it is independent from the sponsor and competing interests; and reference to where further details about its charter can be found, if not in the protocol. Alternatively, an explanation of why a DMC is not needed	Page 6 of the manuscript.
Data monitoring: interim analysis	#21b	Description of any interim analyses and stopping guidelines, including who will have access to these interim results and make the final decision to terminate the trial	Page 6 of the manuscript.
Harms	#22	Plans for collecting, assessing, reporting, and managing solicited and spontaneously reported adverse events and other unintended effects of trial interventions or trial conduct	Page 5 of the manuscript.
Auditing	#23	Frequency and procedures for auditing trial conduct, if any, and whether the process will be independent from investigators and the sponsor	Page 6 of the manuscript.

Ethics and dissemination

Research ethics approval	#24	Plans for seeking research ethics committee / institutional review board (REC / IRB) approval	Page 6 of the manuscript.

For peer review only

Protocol amendments	#25	Plans for communicating important protocol modifications (eg, changes to eligibility criteria, outcomes, analyses) to relevant parties (eg, investigators, REC / IRBs, trial participants, trial registries, journals, regulators)	Page 6 of the manuscript.
Consent or assent	#26a	Who will obtain informed consent or assent from potential trial participants or authorised surrogates, and how (see Item 32)	Page 6 of the manuscript.
Consent or assent: ancillary studies	#26b	Additional consent provisions for collection and use of participant data and biological specimens in ancillary studies, if applicable	Page 6 of the manuscript.
Confidentiality	#27	How personal information about potential and enrolled participants will be collected, shared, and maintained in order to protect confidentiality before, during, and after the trial	Page 6 of the manuscript.
Declaration of interests	#28	Financial and other competing interests for principal investigators for the overall trial and each study site	Listed on the title page.
Data access	#29	Statement of who will have access to the final trial dataset, and disclosure of contractual agreements that limit such access for investigators	Page 6 of the manuscript.

For peer review only

Ancillary and post trial care	#30	Provisions, if any, for ancillary and post-trial care, and for compensation to those who suffer harm from trial participation	Page 6 of the manuscript.
Dissemination policy: trial results	#31a	Plans for investigators and sponsor to communicate trial results to participants, healthcare professionals, the public, and other relevant groups (eg, via publication, reporting in results databases, or other data sharing arrangements), including any publication restrictions	Page 6 of the manuscript.
Dissemination policy: authorship	#31b	Authorship eligibility guidelines and any intended use of professional writers	Listed on the title page.
Dissemination policy: reproducible research	#31c	Plans, if any, for granting public access to the full protocol, participant-level dataset, and statistical code	Page 7 of the manuscript.

Appendices

Informed consent materials	#32	Model consent form and other related documentation given to participants and authorised surrogates	Page 6 of the manuscript.
Biological specimens	#33	Plans for collection, laboratory evaluation, and storage of biological specimens for genetic or molecular analysis in the current trial and for future use in ancillary studies, if applicable	n/a Biological specimens are none.

For peer review only

None The SPIRIT Explanation and Elaboration paper is distributed under the terms of the Creative Commons Attribution License CC-BY-NC. This checklist can be completed online using https://www.goodreports.org/, a tool made by the EQUATOR Network in collaboration with Penelope.ai

For peer review only

BMJ Open

Randomised controlled trial on the effect of video-conference cognitive behavioural therapy for patients with schizophrenia: A study protocol

Journal:	*BMJ Open*
Manuscript ID	bmjopen-2022-069734.R1
Article Type:	Protocol
Date Submitted by the Author:	20-Jul-2023
Complete List of Authors:	Katsushima, Masayuki; Chiba University Graduate School of Medicine School of Medicine, Departments of Cognitive Behavioral Physiology; Teikyo Heisei University - Chiba Campus, Department of Rehabilitation, Faculty of Health Care and Medical Sports<br>Nakamura, Hideki; Chiba University Graduate School of Medicine School of Medicine, Departments of Cognitive Behavioral Physiology; Jikei University School of Medicine, Department of Nursing, Faculty of Medicine<br>Hanaoka, Hideki; Chiba University, Clinical Research Center<br>Shiko, Yuki; Chiba University Hospital, Clinical Research Center; Chiba University,<br>Komatsu, Hideki; Kameda Medical Center, Department of Psychiatry and Psychosomatic Medicine<br>Shimizu, Eiji; Chiba University, Department of Cognitive Behavioral Physiology, Graduate School of Medicine; Chiba University, Research Center for Child Mental Development, Graduate School of Medicine
**Primary Subject Heading**:	Mental health
Secondary Subject Heading:	Mental health, Rehabilitation medicine
Keywords:	Schizophrenia & psychotic disorders < PSYCHIATRY, MENTAL HEALTH, Telemedicine < BIOTECHNOLOGY & BIOINFORMATICS

SCHOLARONE™
Manuscripts

Randomised controlled trial on the effect of video-conference cognitive behavioural therapy for patients with schizophrenia: A study protocol

For peer review only

For peer review only - http://bmjopen.bmj.com/site/about/guidelines.xhtml

ABSTRACT

Introduction Cognitive behavioural therapy for psychosis (CBTp) has demonstrated effectiveness in reducing positive symptoms, improving depression, enhancing coping skills, and increasing awareness of illness. However, compared to cognitive behavioural therapy for depression and anxiety, the spread of CBTp in clinical practice is minimal. The present study designed a randomised controlled trial (RCT) research protocol to evaluate whether real-time remote video-conference CBTp (vCBTp) could facilitate access to psychosocial interventions and effectively improve symptoms compared to usual care (UC) for patients with schizophrenia.

Methods and analysis This exploratory RCT will consist of two parallel groups (vCBTp+UC and UC alone) of 12 participants (n=24) diagnosed with schizophrenia, schizoaffective disorder, or paranoid disorder, who remain symptomatic following pharmacotherapy. Seven 50-minute weekly vCBTp interventions will be administered to test efficacy. The primary outcome will be the positive and negative syndrome scale (PANSS) score at week 8. The secondary outcome will be the Beck Cognitive Insight Scale to assess insight, the Patient Health Questionnaire-9 (PHQ-9) to assess depression, the Generalized Anxiety Disorder-7 (GAD-7) to assess anxiety, the 5-level EuroQol 5-dimensional questionnaire (EQ-5D-5L) to assess quality of life, and the impact of Event Scale-Revised (IES-R) to assess subjective distress about a specific stressful life event. We will take all measurements at 0 weeks (baseline) and at eight weeks (post-intervention), and apply intention-to-treat analysis.

Ethics and dissemination We will conduct this study in the outpatient department of Cognitive Behavioral Therapy Center at Chiba University Hospital. Further, all participants will be informed of the study and will be asked to sign consent forms. We will report according to the Consolidated Standards of Reporting Trials (CONSORT).

Trial registration number The clinical trial registration number is UMIN000043396.

Strengths and limitations of this study

►This study will be a pilot randomised controlled trial to test the effectiveness of real-time remote cognitive behavioral therapy using a video-conference system for outpatients diagnosed with schizophrenia.

►The intervention comprises seven sessions, with elements of psychoeducation, case

For peer review only - http://bmjopen.bmj.com/site/about/guidelines.xhtml

formulation, and cognitive restructuring.

► Remote cognitive behavioral therapy in this study has the potential to reduce the burden of access for receiving cognitive behavioral therapy for schizophrenia.

► This is a single-center pilot study, and statistical analysis between the UC and vCBTp+UC groups will be limited to exploratory comparisons.

For peer review only

For peer review only - http://bmjopen.bmj.com/site/about/guidelines.xhtml

INTRODUCTION

Based on the fifth edition of the Diagnostic and Statistical Manual of Mental Disorders (DSM-5), schizophrenia is a mental disorder associated with functional disability, poor quality of life, and early death.(1) Schizophrenia is a severe, lifelong mental disorder that affects approximately 1% of the world's population.(2) The illness tends to develop between the ages of 16 and 30 years.(3) Individuals with schizophrenia use a substantial amount of healthcare services. This condition imposes a significant economic burden on both the patients and their families, and on society as a whole.(4)

Standard treatment for schizophrenia

The treatment of schizophrenia is multi-dimensional and comprehensive. The first treatment option is pharmacotherapy with antipsychotic medications and psychological interventions, such as cognitive-behavioral therapy, as recommended by the National Institute of Health and Clinical Excellence.(5) Antipsychotic medications are commonly used for patients with schizophrenia primarily to reduce positive symptoms, such as hallucinations and delusions by regulating the activity of dopamine and other nerves in the brain. In recent years, atypical antipsychotic prescriptions have been promoted. They effectively improve positive and negative symptoms and cognitive function but may cause side effects in some patients.

The American Psychiatric Association guidelines,(6) and the Schizophrenia Patient Outcomes Research Team,(7) recommend psychosocial interventions in combination with pharmacotherapy. Comprehensive treatment measures include individual and group psychotherapy, psychoeducation, occupational therapy, family therapy, and psychosocial interventions such as social skills training.(8)

Cognitive behavioural therapy for schizophrenia

Cognitive behavioural therapy for psychosis (CBTp) has been shown to reduce positive symptoms and improve coping skills in Europe and the United States.(9) Kuller *et al*(10) reported that CBTp is used in 58% of U.S. medical facilities and 91.3% of U.K. medical facilities. Wykes *et al*(11) reported an effect size of 0.40 (0.25-0.55) in a meta-analysis of 33 RCTs (n=1,964) on CBTp. Jauhar *et al*(12) found an effect size of 0.33 for general symptoms in a meta-analysis of 34 studies on CBTp for schizophrenia. The National Institute of Health and Clinical Excellence recommends a session structure of 16 sessions or more(5). However, despite the evidence, the number of practitioners is insufficient; thus, it is challenging for patients to access CBTp.(13) Additionally, CBTp is important because patients' characteristics and problems are diverse due to the illness's nature. It is essential to be flexible in dealing with mood and thoughts, realistic and rational thoughts, and delusional and pathological experiences.(14)

There are reports of an association between intrusive thoughts of post-traumatic experiences and

For peer review only - http://bmjopen.bmj.com/site/about/guidelines.xhtml

schizophrenia.(15),(16) There is also a discussion of the potential for CBTp to address intrusive thoughts.(17)

In the future, it will be desirable to establish an effective implementation system for CBTp and increase the number of practitioners in clinical practice.

Evidence of low-intensity CBTp has been collected overseas, and its effectiveness has been recognised, too.(18),(19) Turkington *et al*(20),(21) found that community psychiatric nurses' provision of low-intensity CBTp, comprising six sessions, effectively increased awareness of the illness. Overall, six sessions of a six-week low-intensity CBTp provided by community psychiatric nurses improved the understanding of illness, general status, and depression. This suggests that CBTp offered by health care professionals may also be effective. Recently, studies have begun investigating CBTp that can be effectively administered online using the Internet.(22–25)

Video-conference cognitive behavioural therapy for psychosis

The number of CBTp practitioners is limited, and their use in clinical practice is much less frequent. As a remedy to this problem, there is growing interest in using video-conferenced systems to provide CBT, known as vCBT.(26) This approach has the advantage of providing access to CBT for patients living in remote areas and enabling remote treatment through interactive, real-time communication between the therapist and patient.(27)Although therapist guided Internet-based CBT is shown to be not inferior to face-to-face CBT in treating anxiety, depression, insomnia and somatic disorders,(28) there is less reports about Internet-based CBTp. Up to this point in 2022, to the best of our knowledge, no studies have examined the efficacy of individual CBTp using a video-conference system (vCBTp) for schizophrenia.(29)

Objective

This article describes the study protocol of a randomised controlled trial designed to evaluate the clinical efficacy of seven sessions of vCBTp, as an adjunct to usual care (UC) compared to UC alone. The trial focuses on patients with schizophrenia who continue experiencing positive symptoms despite receiving pharmacotherapy.

METHODS AND ANALYSIS

Study design

The study is designed as a single center, assessor-blind, two-arm, parallel, prospective, randomised, controlled trial comprising a 7-week treatment regimen. Participants will be allocated to vCBTp plus UC or UC alone (Figure 1). We will report according to the Consolidated Standards of Reporting Trials (CONSORT) and the SPIRIT reporting guidelines.(30)

For peer review only - http://bmjopen.bmj.com/site/about/guidelines.xhtml

Participants

Inclusion criteria of participants in this study are between 16 and 65 years of age, primary diagnosis of schizophrenia, schizoaffective disorder, or delusional disorder according to the fifth edition of the Diagnostic and Statistical Manual of Mental Disorders (DSM-5), and who are found competent to consent to the study in the MacArthur Competence Assessment Tool for Clinical Research (MacCAT-CR),(31,32) having at least three out of seven points on any one of the seven positive and negative syndrome scale (PANSS) positive symptoms, and those who have taken antipsychotic drugs constantly for 3 months or more and are not planning to change their drugs for the next 3 months. In addition, participants will be required to have Internet and computer access and an environment that allowed them to take the vCBTp. The exclusion criteria are severe addiction and substance dependence, such as alcohol or drug dependence, intellectual disability, neurocognitive disorders, or predicted risk of self-harm or other harm due to worsening symptoms reported by the participants, their family members, or their psychiatrists. The risk will be assessed at the time of obtaining consent and at each endpoint by responses to the outcome item and by observation of adverse events. Specifically, patients will be screened for suicidal ideation with nine of the PHQ-9. In particular, those at risk of imminent suicide and are expected to discontinue vCBTp. And those who are hospitalised.

Two researchers (a psychiatrist [ES] and a researcher [MK]) will evaluate and confirm the patients' eligibility including diagnosis, treatment history by their psychiatrists, the suitability of the symptoms and their ability to consent to participate in the study.

Details of recruitment

The researchers will recruit participants through websites, posters and leaflets placed at medical institutions in Chiba Prefecture, Japan, from April 2021 to March 2025 until 24 participants will be enrolled. Participants will be required to obtain permission from their psychiatrists before enrollment in the study, and continue to receive medical care including pharmacotherapy from their psychiatrists. The study will be conducted in the Cognitive Behavioural Therapy Center outpatient clinic at Chiba University Hospital.

Intervention methods

Participants will not be restricted to changes in their medications by their psychiatrists during the study period. If clinically appropriate, they will be allowed to participate in usual psychosocial intervention programs, such as social skills training at psychiatric daycare or night care.

However, to properly evaluate the effectiveness of vCBTp, participants may not receive other cognitive behavioural therapy or specific psychotherapies including psychoanalytic therapy, Morita therapy, insight therapy, hypnotherapy, brain stimulation therapies including electroconvulsive

For peer review only - http://bmjopen.bmj.com/site/about/guidelines.xhtml

therapy or magnetic stimulation therapies. The vCBTp+UC group will receive the text material developed by the research team by mail. All seven sessions will be conducted according to the text material. The therapist will assign homework to the participants at the end of each session, as described in the text material.

Video-conference cognitive behavioral therapy program for schizophrenia

The vCBTp program for schizophrenia was developed by two researchers (ES and MK). The number of sessions was set at seven, as the median number of sessions was 7.5 in a meta-analysis of low-intensity CBTp.(18) The treatment lasts over seven weeks (once a week, 50 minutes per session). It included elements incorporated into CBT for depression and anxiety, in general. This protocol primarily focuses on cognitive restructuring. The components are as follows: (1) Assessment and goal setting, (2) externalization of stress in present life, (3) cognitive restructuring of stress in present life, (4) mood change and relaxation, (5) externalization of past stressful experiences, (6) reappraisal of past stressful experiences, and (7) relapse prevention. As described in the introduction, we focus on subjective distress from intrusive thoughts of stressful experiences in patients with schizophrenia.(15–17) We allowed flexibility on changing or repeating the order of sessions depending on participants' understanding and reactions.

Each session includes examples of cognitive restructuring for patients with schizophrenia and homework assignments. The therapist conducting the session and the participant will be connected remotely in real-time via a web conferencing system between the participant's house and Chiba University Hospital for cognitive-behavioural therapy. Participants will have to work on the vCBTp and receive supportive feedback on the requested homework. Participants will also be allowed to email the research office (MK) if they have questions regarding the contents.

Outcomes

Baseline and clinical characteristics

The baseline and clinical characteristics included sex, age, marital status, employment status, age at onset of schizophrenia, duration of illness and treatment history including the name of the antipsychotic to which the patient has developed resistance, current drug titers at baseline, and any changes in conventional treatment during the study period will be collected by researchers.

Primary outcome

The primary outcome is change in the PANSS(33) total score at week 8 from baseline, The PANSS is a 30-item interview-based assessment consisting of seven positive symptoms, seven negatives, and 16 general psychopathology items. Each subscale is scored on a scale of 1-7 points. The PANSS is an objective scale for assessing schizophrenia symptoms in clinical and experimental studies and

For peer review only - http://bmjopen.bmj.com/site/about/guidelines.xhtml

has become a worldwide standard for reliability and validity. An assessor (a researcher, HN) blind to treatment allocation will be evaluated the PANSS by web conferencing system at baseline and at week 8, respectively. Comparative results between video-conference and face-to-face assessments for schizophrenia are similar.(34) Furthermore, it has been suggested that there is no difference between face-to-face and remote assessments on the PANSS.(35)

Secondary outcomes

The secondary outcomes are the following;

We will evaluate the sum of positive symptoms, negative symptoms, and general psychopathology extracted from the PANSS subscales score, respectively. The total score of the seven positive symptoms subscale on the PANSS ranges from 7 (no symptoms) to 49 (severe positive symptoms). The total score of the seven negative symptoms subscale ranges from 7 (no symptoms) to 49 (severe negative symptoms). The total score of the 16 items of general psychopathology subscale ranged from 16 (no symptoms) to 112 (severity of psychopathology).

The Japanese version of the Beck Cognitive Insight Scale (BCIS-J)(36) will be used to assess cognitive insight. A total of 15 items of the BCIS-J are divided into nine items of self-reflection (0-36) and six items of self-confidence (0-24). The lower the subtotal of nine self-reflection items minus six self-confidence items, the lower the cognitive pathology.

The Impact of Event Scale-Revised (IES-R)(37) will be used to assess subjective distress for a specific stressful life event. The 22 items (0-88) of IES-R comprise eight intrusive symptoms, eight avoidance symptoms, and six hyperarousal symptoms, and can measure symptoms in persons exposed to traumatic experiences.

The Patient Health Questionnaire-9 (PHQ-9)(38) will be used to assess depressive symptoms. PHQ-9 comprise nine-item ranges from 0 (no depressive symptoms) to 27 (severe depressive symptoms).

The Generalized Anxiety Disorder-7(GAD-7)(39) will be used to assess anxiety. GAD-7 comprise seven-item ranged from 0 (no anxiety symptoms) to 21 (severe anxiety symptoms).

The EuroQol five-dimensional questionnaire (EQ-5D-5L)(40) will be used to assess QOL. It comprises five items and assesses the quality of life on a five-point Likert scale from 1 (not severe) to 5 (severe). It is the most commonly used measure of quality-adjusted life-years worldwide.

In addition, we will evaluate the chlorpromazine equivalent to daily prescriptions for antipsychotic medications.(41)

Therapists will ask the participants about their experience of adverse events during each assessment. All measures will be assessed at week 0 (baseline) and week 8 (post-intervention), and the results will be analyzed per the intention-to-treat principle.

Sample size

For peer review only - http://bmjopen.bmj.com/site/about/guidelines.xhtml

Primarily, this study aims to test the superiority of vCBTp on symptom improvement in a group of patients with schizophrenia compared to a usual care UC group. We refer to a previous study by Morrison *et al*(42) in which CBT and antipsychotic groups were compared. Assuming that the mean difference between the vCBTp plus UC group and UC alone group after the intervention is 12 points and the standard deviation of the pooled UC group is eight, the number of patients in each group was calculated as 11 for a significance level of 5% on both sides and a power of 90%. Therefore, the target number of patients was set at 12 in each group, assuming a dropout rate of 10% for 24 patients. Furthermore, a previous study suggested that 12 patients in each group is an appropriate number in a pilot study.(43)

Randomisation and assessor-blindness

After baseline assessment, participants will be randomly assigned to the UC group or vCBTp+UC in a 1:1 ratio, using the minimization method to ensure a balance between baseline PANSS total score (PANSS≥51) and sex. The PANSS values are based on a study by Naeem *et al*(44) Each participant will be randomly assigned to one of the two treatments. The assessor (a researcher, HN) will be not informed of the participant's allocation group throughout the study period for assessor-blindness.

Data analysis plan

The study will follow the CONSORT guidelines for statistical analysis and reporting. The primary analysis will be based on the intention-to-treat principle. To evaluate the primary outcome, the changes in the PANSS total score at week 8 will be calculated for both the UC group and the vCBTp+UC group. A comparison between the groups will be performed using an unpaired t-test. Secondary outcomes will also be analyzed to provide further understanding of the primary endpoints, following a similar approach as the primary outcome analysis. In addition, the number of subjects in each group who have improved by 20% of the total PANSS score are considered to be treatment-responsive, and the proportion of subjects in each group is calculated. The $\chi 2$ test is used to compare proportions between groups. The safety endpoint is the frequency of adverse events, and a tabulation table is prepared for the endpoints, with exact two-sided 95% confidence intervals of the binomial distribution calculated for each group for percentage estimates. If necessary, a comparison between groups is made using the Fisher's direct probability calculation method. The statistical analyses will be performed using SAS ver. 9.4 (SAS Institute Inc., Cary, NC, USA), and a p-value of less than 0.05 will be considered statistically significant.

Ethics and dissemination

The study protocol has been approved by the Institutional Review Board of the Chiba University

For peer review only - http://bmjopen.bmj.com/site/about/guidelines.xhtml

Hospital (reference number: G2020031) on January 2021. The clinical trial registration number is UMIN000043396.

We will conduct this study at the Outpatient Department of the Cognitive Behavioural Therapy Center at Chiba University Hospital. Prospective participants will be informed of the purpose of the study and asked about their willingness to participate when they contact the researchers. Participation is voluntary and complete anonymity will be guaranteed. Further, participants would be notified that they could drop out of the study at any point. Each participant will be asked to provide written informed consent to participate in the study after MacCAT-CR confirms that the participant is competent to consent to the clinical trial. All participants will receive UC from their psychiatrists, and half of the participants will receive vCBTp in addition to UC. Participants randomised to the UC arm will be eligible to receive vCBTp in an ancillary study (UMIN000044244) after completion of the study. A blinded assessor will evaluate the participants at each assessment point (Weeks 0 and 8). All adverse events will be reported, and serious ones will be immediately reported to the Clinical Trial Review Committee of Chiba University Hospital. Moreover, they will be registered in the hospital's risk management system. An independent data monitoring committee then properly review detailed records of the progress of the clinical trial, key efficacy variables, and safety data and recommend the trial's continuation, modification, or termination to the clinical investigators accordingly. Regardless of the outcome, the trial results will be published in an international journal. This study will be conducted and reported per Consolidated Standards of Reporting Trials (CONSORT) recommendations.

Patient and public involvement

Patients and the public were not involved in the design, or conduct of the study.

DISCUSSION

This study will address the effect of vCBTp for patients with schizophrenia who continue experiencing positive symptoms despite receiving pharmacotherapy.(13,20,21,45,46) The findings of this study will provide valuable evidence to facilitate the development of new therapeutic support modalities and promote increased treatment options for patients. The anticipated limitations of this study are as follows. First, because this study is a single-center study, it has less generalizability than those conducted in multi-center studies. Second, the follow-up study is set up as a separate study. Therefore, verifying the retention of this effect in this study is not easy. The present study is a pilot study, and the study design is based on consideration of patient burden. If this study is successfully completed without any serious adverse events resulting in hospitalization or potentially leading to disability, and if a certain level of efficacy is suggested, it would be advisable to plan a full-scale study with a larger sample size.

For peer review only - http://bmjopen.bmj.com/site/about/guidelines.xhtml

Title of this article

Randomised controlled trial on the effect of video-conference cognitive behavioural therapy for patients with schizophrenia: A study protocol

Author name

Masayuki Katsushima[1,2] , Hideki Nakamura[1,3] , Hideki Hanaoka[4] , Yuki Shiko[4] , Hideki Komatsu[5] , Eiji Shimizu[1,6,7]

Author affiliations

[1] Departments of Cognitive Behavioral Physiology, Chiba University Graduate School of Medicine, Chiba, Japan

[2] Department of Rehabilitation, Faculty of Health Care and Medical Sports, Teikyo Heisei University, Chiba, Japan

[3] Department of Nursing, Faculty of Medicine, The Jikei University School of Medicine, Tokyo, Japan

[4] Clinical Research Center, Chiba University Hospital, Chiba, Japan

[5] Department of Psychiatry and Psychosomatic Medicine, Kameda Medical Center, Chiba, Japan

[6] Cognitive Behavioral Therapy Center, Chiba University Hospital, Chiba, Japan

[7] Research Center for Child Mental Development, Chiba University, Chiba, Japan

Acknowledgements

We thank all the staff at the Cognitive Behavioral Therapy Center of Chiba University Hospital. We are grateful to all participants in this study. We would like to thank Editage (www.editage.com) for English language editing.

Contact address of corresponding author

Masayuki Katsushima[1,2]

[1] 1-8-1 Inohana, Chuo-ku, Chiba-shi, Chiba, 260-8670 Japan +81-43-226-2027, m.katsushima@thu.ac.jp

Graduate school doctoral course

Co-author: Email address

Hideki Nakamura: hnakamura@jikei.ac.jp

Hideki Hanaoka: hanaoka.hideki@mac.com

Yuki Shiko: shiko_yuki@chiba-u.jp

For peer review only - http://bmjopen.bmj.com/site/about/guidelines.xhtml

Hideki Komatsu: chibakomatsu@gmail.com

Eiji Shimizu: eiji@faculty.chiba-u.jp

Contributors

MK and ES designed the study and developed the protocol with HH and YS; MK, ES, and HK recruited the patients; MK, HN, and ES managed the preparation of the manuscript; MK wrote the first draft of the manuscript; MK, HN, and ES wrote the manuscript. All the authors contributed to and approved the final version of the manuscript.

Funding

This work was supported by a Grant-in-Aid for Scientific Research (Grant No. 21K11197, Japan Society for the Promotion of Science).

Competing interests

None declared.

Patient consent

Obtained

Data sharing statement

Data sharing is not applicable to this article as no datasets were generated or analyzed during the current study.

Ethics approval

The study protocol has been approved by the Institutional Review Board of the Chiba University Hospital (reference number: G2020031) on January 2021. The clinical trial registration number is UMIN000043396.

English proofreading

Editage (www.editage.com)

Total word count of the article

3274 words. (Title, Abstract and Manuscript)

Number of figures and tables

1 figure.

For peer review only

keyword

①Schizophrenia ②Cognitive Behavioural Therapy ③Remote ④Accessibility

For peer review only

For peer review only - http://bmjopen.bmj.com/site/about/guidelines.xhtml

REFERENCES

1. Laursen TM, Nordentoft M, Mortensen PB. Excess early mortality in schizophrenia. Annu Rev Clin Psychol. 2014 Mar 10:425-448. doi/10.1146/annurev-clinpsy-032813-153657

2. Saha S, Chant D, Welham J, et al. A Systematic Review of the Prevalence of Schizophrenia. *PLoS Med.* 2005 May 31;2(5): e141. doi: 10.1371/journal.pmed.0020141

3. Mueser KT, McGurk SR. Schizophrenia. The Lancet. 2004 Jun;363(9426):2063–72. doi: 10.1016/S0140-6736(04)16458-1

4. Kovács G, Almási T, Millier A, et al. Direct healthcare cost of schizophrenia – European overview. Eur Psychiatry. 2018;48(1):79–92. doi: 10.1016/j.eurpsy.2017.10.008

5. Psychosis and schizophrenia in adults: prevention and management. London: National Institute for Health and Care Excellence (NICE); 2014. (National Institute for Health and Care Excellence: Guidelines). Available from: http://www.ncbi.nlm.nih.gov/books/NBK555203/ PMID: 32207892

6. Keepers GA, Fochtmann LJ, Anzia JM, et al. The American Psychiatric Association Practice Guideline for the Treatment of Patients With Schizophrenia. FOCUS. 2020 Oct;18(4):493–7. doi: 10.1176/appi.focus.18402

7. Dixon LB, Dickerson F, Bellack AS, et al. The 2009 Schizophrenia PORT Psychosocial Treatment Recommendations and Summary Statements. Schizophr Bull. 2010 Jan 1;36(1):48–70. doi: 10.1093/schbul/sbp115

8. Bighelli I, Rodolico A, García-Mieres H, et al. Psychosocial and psychological interventions for relapse prevention in schizophrenia: a systematic review and network meta-analysis. Lancet Psychiatry. 2021 Nov;8(11):969–80. doi: 10.1016/S2215-0366(21)00243-1

9. Mankiewicz PD, Turner C. Cognitive Restructuring and Graded Behavioural Exposure for Delusional Appraisals of Auditory Hallucinations and Comorbid Anxiety in Paranoid Schizophrenia. Case Rep Psychiatry. 2014;2014:1–8. doi: 10.1155/2014/124564

10. Kuller AM, Ott BD, Goisman RM, et al. Cognitive Behavioral Therapy and Schizophrenia: A Survey of Clinical Practices and Views on Efficacy in the United States and United Kingdom. Community Ment Health J. 2010 Feb;46(1):2–9. doi: 10.1007/s10597-009-9223-6

11. Wykes T, Steel C, Everitt B, et al. Cognitive Behavior Therapy for Schizophrenia: Effect Sizes, Clinical Models, and Methodological Rigor. Schizophr Bull. 2007 May 4;34(3):523–37. doi:

For peer review only - http://bmjopen.bmj.com/site/about/guidelines.xhtml

10.1093/schbul/sbm114

12. Jauhar S, McKenna PJ, Radua J, et al. Cognitive–behavioural therapy for the symptoms of schizophrenia: systematic review and meta-analysis with examination of potential bias. Br J Psychiatry. 2014 Jan;204(1):20–9. doi: 10.1192/bjp.bp.112.116285

13. Shafran R, Clark DM, Fairburn CG, et al. Mind the gap: Improving the dissemination of CBT. Behav Res Ther. 2009 Nov;47(11):902–9. doi: 10.1016/j.brat.2009.07.003

14. Ishigaki T, Kikuchi A, Matsumoto K, et al. Cognitive behavior Therapy for psychosis (CBTp) Learning through Case Studies (in Japanese) 1st ed. Tokyo: Kongo Publishing Co; 2019: 27-33. Available from: https://www.kongoshuppan.co.jp/book/b515016.html

15. Malcolm CP, Picchioni MM, Ellett L. Intrusive prospective imagery, posttraumatic intrusions and anxiety in schizophrenia. Psychiatry Res. 2015 Dec;230(3):899–904. doi: 10.1016/j.psychres.2015.11.029

16. Morrison AP, Baker CA. Intrusive thoughts and auditory hallucinations: a comparative study of intrusions in psychosis. Behav Res Ther. 2000 Nov;38(11):1097–106. doi: 10.1016/S0005-7967(99)00143-6

17. O'Driscoll C, Mason O, Brady F, et al. Process analysis of trauma-focused cognitive behavioural therapy for individuals with schizophrenia. Psychol Psychother Theory Res Pract. 2016 Jun;89(2):117–32. doi: 10.1111/papt.12072

18. Hazell CM, Hayward M, Cavanagh K, et al. A systematic review and meta-analysis of low intensity CBT for psychosis. Clin Psychol Rev. 2016 Apr;45:183–92. doi: 10.1016/j.cpr.2016.03.004

19. Malik N, Kingdon D, Pelton J, et al. Effectiveness of Brief Cognitive-Behavioral Therapy for Schizophrenia Delivered by Mental Health Nurses: Relapse and Recovery at 24 Months. J Clin Psychiatry. 2009 Feb 15;70(2):201–7. doi: 10.4088/JCP.07m03990

20. Turkington D, Kingdon D, Turner T. Effectiveness of a brief cognitive–behavioural therapy intervention in the treatment of schizophrenia. Br J Psychiatry. 2002 Jun;180(06):523–7. doi: 10.1192/bjp.180.6.523

21. Brabban A, Tai S, Turkington D. Predictors of Outcome in Brief Cognitive Behavior Therapy for Schizophrenia. Schizophr Bull. 2009 Sep 1;35(5):859–64. doi: 10.1093/schbul/sbp065

For peer review only

For peer review only - http://bmjopen.bmj.com/site/about/guidelines.xhtml

22. Gottlieb JD, Romeo KH, Penn DL, et al. Web-based cognitive–behavioral therapy for auditory hallucinations in persons with psychosis: A pilot study. Schizophr Res. 2013 Apr;145(1–3):82–7. doi: 10.1016/j.schres.2013.01.002

23. Gottlieb JD, Gidugu V, Maru M, et al. Randomized controlled trial of an internet cognitive behavioral skills-based program for auditory hallucinations in persons with psychosis. Psychiatr Rehabil J. 2017 Sep;40(3):283–92. doi: 10.1037/prj0000258

24. Rüegg N, Moritz S, Berger T, et al. An internet-based intervention for people with psychosis (EviBaS): study protocol for a randomized controlled trial. BMC Psychiatry. 2018 Dec;18(1):102. doi: 10.1186/s12888-018-1644-8

25. Westermann S, Rüegg N, Lüdtke T, et al. Internet-based self-help for psychosis: Findings from a randomized controlled trial. J Consult Clin Psychol. 2020 Oct;88(10):937–50. doi: 10.1037/ccp0000602

26. Matsumoto K, Sutoh C, Asano K, et al. Internet-Based Cognitive Behavioral Therapy With Real-Time Therapist Support via Videoconference for Patients With Obsessive-Compulsive Disorder, Panic Disorder, and Social Anxiety Disorder: Pilot Single-Arm Trial. J Med Internet Res. 2018 Dec 17;20(12): e12091. doi: 10.2196/12091

27. Lecomte T, Abdel - Baki A, Francoeur A, et al. Group therapy via videoconferencing for individuals with early psychosis: A pilot study. Early Interv Psychiatry. 2021 Dec;15(6):1595–601. doi: 10.1111/eip.13099

28. Carlbring P, Andersson G, Cuijpers P, et al. Internet-based vs. face-to-face cognitive behavior therapy for psychiatric and somatic disorders: an updated systematic review and meta-analysis. Cogn Behav Ther. 2018 Jan 2;47(1):1–18. doi: 10.1080/16506073.2017.1401115

29. Kopelovich SL, Turkington D. Remote CBT for Psychosis During the COVID-19 Pandemic: Challenges and Opportunities. Community Ment Health J. 2021 Jan;57(1):30–4. doi: 10.1007/s10597-020-00718-0

30. Chan AW, Tetzlaff JM, Gotzsche PC, et al. SPIRIT 2013 explanation and elaboration: guidance for protocols of clinical trials. BMJ. 2013 Jan 9;346(jan08 15):e7586–e7586. doi: 10.1136/bmj.e7586

31. Hostiuc S, Rusu MC, Negoi I, et al. Testing decision-making competency of schizophrenia participants in clinical trials. A meta-analysis and meta-regression. BMC Psychiatry. 2018 Dec

For peer review only - http://bmjopen.bmj.com/site/about/guidelines.xhtml

18(1):2. doi: 10.1186/s12888-017-1580-z

32. Fovet T, Amad A, Thomas P, et al. Schizophrénie et consentement à la recherche biomédicale. L'Encéphale. 2015 Oct;41(5):394–402. doi: 10.1016/j.encep.2014.07.004

33. Kay SR, Fiszbein A, Opler LA. The Positive and Negative Syndrome Scale (PANSS) for Schizophrenia. Schizophr Bull. 1987 Jan 1;13(2):261–76. doi: 10.1093/schbul/13.2.261

34. Sharp IR, Kobak KA, Osman DA. The use of videoconferencing with patients with psychosis: a review of the literature. Ann Gen Psychiatry. 2011;10(1):14. doi: 10.1186/1744-859X-10-14

35. Targum SD, Pendergrass JC, Murphy C. Audio-digital recordings to assess ratings reliability in clinical trials of schizophrenia. Schizophr Res. 2021 Jun;232:54–60. doi: 10.1016/j.schres.2021.05.004

36. Uchida T, Matsumoto K, Kikuchi A, et al. Psychometric properties of the Japanese version of the Beck Cognitive Insight Scale: Relation of cognitive insight to clinical insight: Cognitive insight in schizophrenia. Psychiatry Clin Neurosci. 2009 Jun;63(3):291–7. doi: 10.1111/j.1440-1819.2009.01946.x

37. Asukai N, Kato H, Kawamura N, et al. Rellability and validity of the Japanese-language version of the Impact of Event Scale-Revised (IES-R-J): four studies of different traumatic events: J Nerv Ment Dis. 2002 Mar;190(3):175–82. doi: 10.1097/00005053-200203000-00006

38. Muramatsu K, Kamijima K, Yoshida M, et al. The Patient Health Questionnaire, Japanese Version: Validity According to the Mini-International Neuropsychiatric Interview–Plus. Psychol Rep. 2007 Dec;101(3):952–60. doi: 10.2466/pr0.101.3.952-960

39. Spitzer RL, Kroenke K, Williams JBW, et al. A Brief Measure for Assessing Generalized Anxiety Disorder: The GAD-7. Arch Intern Med. 2006 May 22;166(10):1092. doi: 10.1001/archinte.166.10.1092

40. Feng YS, Kohlmann T, Janssen MF, et al. Psychometric properties of the EQ-5D-5L: a systematic review of the literature. Qual Life Res. 2021 Mar;30(3):647–73. doi: 10.1007/s11136-020-02688-y

41. Woods SW. Chlorpromazine Equivalent Doses for the Newer Atypical Antipsychotics. J Clin Psychiatry. 2003 Jun 15;64(6):663–7. doi: 10.4088/JCP.v64n0607

42. Morrison AP, Law H, Carter L, et al. Antipsychotic drugs versus cognitive behavioural therapy

For peer review only - http://bmjopen.bmj.com/site/about/guidelines.xhtml

versus a combination of both in people with psychosis: a randomised controlled pilot and feasibility study. Lancet Psychiatry. 2018 May;5(5):411–23. doi: 10.1016/S2215-0366(18)30096-8

43. Julious SA. Sample size of 12 per group rule of thumb for a pilot study. Pharm Stat. 2005 Oct;4(4):287–91. doi: 10.1002/pst.185

44. Naeem F, Johal R, McKenna C, et al. Cognitive Behavior Therapy for psychosis based Guided Self-help (CBTp-GSH) delivered by frontline mental health professionals: Results of a feasibility study. Schizophr Res. 2016 May;173(1–2):69–74. doi: 10.1016/j.schres.2016.03.003

45. Pillny M, Lincoln TM. Moderne kognitive Verhaltenstherapie bei psychotischen Störungen. Nervenarzt. 2020 Jan;91(1):43–9. doi 10.1007/s00115-019-00831-w

46. Jolley S, Onwumere J, Kuipers E, et al. Increasing access to psychological therapies for people with psychosis: Predictors of successful training. Behav Res Ther. 2012 Aug;50(7–8):457–62. doi: 10.1016/j.brat.2012.04.002

Figure legend: CONSORT patients' flow diagram of the study, randomisation and treatment. CONSORT, Consolidated Standards of Reporting Trials.

Figure1 – Flow chart

For peer review only - http://bmjopen.bmj.com/site/about/guidelines.xhtml

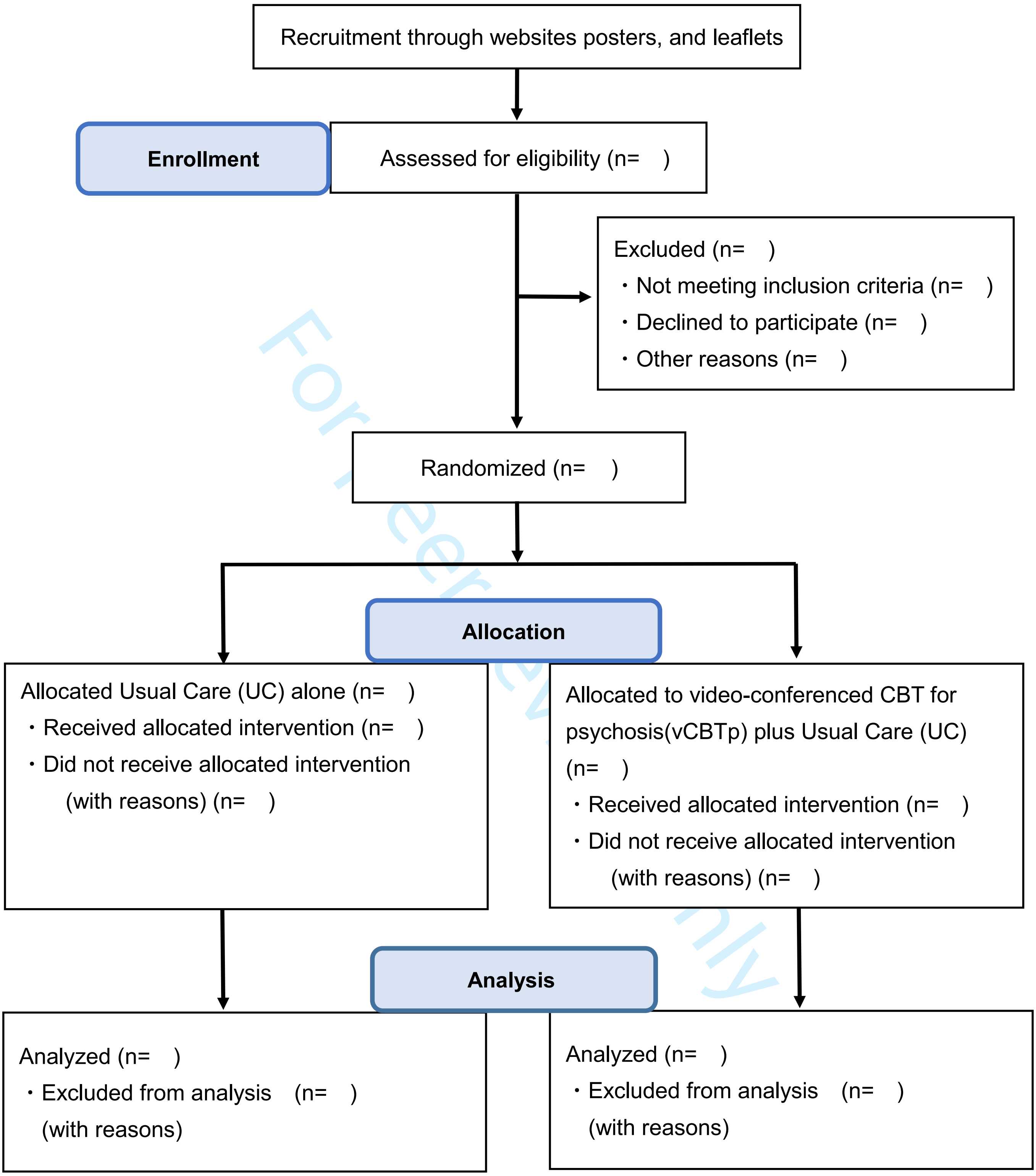
Recruitment through websites posters, and leaflets
Enrollment
Assessed for eligibility (n=)
Excluded (n=)
・Not meeting inclusion criteria (n=)
・Declined to participate (n=)
・Other reasons (n=)
Randomized (n=)
Allocation
Allocated Usual Care (UC) alone (n=)
・Received allocated intervention (n=)
・Did not receive allocated intervention (with reasons) (n=)
Allocated to video-conferenced CBT for psychosis(vCBTp) plus Usual Care (UC) (n=)
・Received allocated intervention (n=)
・Did not receive allocated intervention (with reasons) (n=)
Analysis
Analyzed (n=)
・Excluded from analysis (n=) (with reasons)
Analyzed (n=)
・Excluded from analysis (n=) (with reasons)

Reporting checklist for protocol of a clinical trial.

Based on the SPIRIT guidelines.

Instructions to authors

Complete this checklist by entering the page numbers from your manuscript where readers will find each of the items listed below.

Your article may not currently address all the items on the checklist. Please modify your text to include the missing information. If you are certain that an item does not apply, please write "n/a" and provide a short explanation.

Upload your completed checklist as an extra file when you submit to a journal.

In your methods section, say that you used the SPIRITreporting guidelines, and cite them as:

Chan A-W, Tetzlaff JM, Gøtzsche PC, Altman DG, Mann H, Berlin J, Dickersin K, Hróbjartsson A, Schulz KF, Parulekar WR, Krleža-Jerić K, Laupacis A, Moher D. SPIRIT 2013 Explanation and Elaboration: Guidance for protocols of clinical trials. BMJ. 2013;346:e7586

For peer review only

		Reporting Item	Page Number
**Administrative information**			
Title	#1	Descriptive title identifying the study design, population, interventions, and, if applicable, trial acronym	Listed on page1 and the title page.

Trial registration	#2a	Trial identifier and registry name. If not yet registered, name of intended registry	Page 2 of the manuscript.
Trial registration: data set	#2b	All items from the World Health Organization Trial Registration Data Set	n/a
Protocol version	#3	Date and version identifier	n/a
Funding	#4	Sources and types of financial, material, and other support	Listed on page 12
Roles and responsibilities: contributorship	#5a	Names, affiliations, and roles of protocol contributors	Listed on page 12
Roles and responsibilities: sponsor contact information	#5b	Name and contact information for the trial sponsor	n/a<br>Competing interests are none.
Roles and responsibilities: sponsor and funder	#5c	Role of study sponsor and funders, if any, in study design; collection, management, analysis, and interpretation of data; writing of the report; and the decision to submit the report for publication, including whether they will have ultimate authority over any of these activities	n/a<br>Competing interests are none.
Roles and responsibilities: committees	#5d	Composition, roles, and responsibilities of the coordinating centre, steering committee, endpoint adjudication committee, data management team,	Page 9-10 of the manuscript.

For peer review only

		and other individuals or groups overseeing the trial, if applicable (see Item 21a for data monitoring committee)	
**Introduction**			
Background and rationale	#6a	Description of research question and justification for undertaking the trial, including summary of relevant studies (published and unpublished) examining benefits and harms for each intervention	Page 4 of the manuscript.
Background and rationale: choice of comparators	#6b	Explanation for choice of comparators	Page 5 of the manuscript.
Objectives	#7	Specific objectives or hypotheses	Page 5 of the manuscript.
Trial design	#8	Description of trial design including type of trial (eg, parallel group, crossover, factorial, single group), allocation ratio, and framework (eg, superiority, equivalence, non-inferiority, exploratory)	Page 5 of the manuscript.
**Methods: Participants, interventions, and outcomes**			
Study setting	#9	Description of study settings (eg, community clinic, academic hospital) and list of countries where data	Page 6 of the manuscript.

For peer review only

		will be collected. Reference to where list of study sites can be obtained	
Eligibility criteria	#10	Inclusion and exclusion criteria for participants. If applicable, eligibility criteria for study centres and individuals who will perform the interventions (eg, surgeons, psychotherapists)	Page 6 of the manuscript.
Interventions: description	#11a	Interventions for each group with sufficient detail to allow replication, including how and when they will be administered	Page 6 of the manuscript.
Interventions: modifications	#11b	Criteria for discontinuing or modifying allocated interventions for a given trial participant (eg, drug dose change in response to harms, participant request, or improving / worsening disease)	Page 6 of the manuscript.
Interventions: adherance	#11c	Strategies to improve adherence to intervention protocols, and any procedures for monitoring adherence (eg, drug tablet return; laboratory tests)	Page 6 of the manuscript.
Interventions: concomitant care	#11d	Relevant concomitant care and interventions that are permitted or prohibited during the trial	Page 6 of the manuscript.
Outcomes	#12	Primary, secondary, and other outcomes, including the specific measurement variable (eg, systolic blood pressure), analysis metric (eg, change from baseline, final value, time to event), method of aggregation (eg, median, proportion), and time point for each outcome. Explanation of the clinical	Page 7-8 of the manuscript.

For peer review only

		relevance of chosen efficacy and harm outcomes is strongly recommended	
Participant timeline	#13	Time schedule of enrolment, interventions (including any run-ins and washouts), assessments, and visits for participants. A schematic diagram is highly recommended (see Figure)	Page 5 of the manuscript.
Sample size	#14	Estimated number of participants needed to achieve study objectives and how it was determined, including clinical and statistical assumptions supporting any sample size calculations	Page 9 of the manuscript.
Recruitment	#15	Strategies for achieving adequate participant enrolment to reach target sample size	Page 6 of the manuscript.
**Methods: Assignment of interventions (for controlled trials)**			
Allocation: sequence generation	#16a	Method of generating the allocation sequence (eg, computer-generated random numbers), and list of any factors for stratification. To reduce predictability of a random sequence, details of any planned restriction (eg, blocking) should be provided in a separate document that is unavailable to those who enrol participants or assign interventions	Page 5 of the manuscript.

For peer review only

Allocation concealment mechanism	#16b	Mechanism of implementing the allocation sequence (eg, central telephone; sequentially numbered, opaque, sealed envelopes), describing any steps to conceal the sequence until interventions are assigned	Page 5 of the manuscript.
Allocation: implementation	#16c	Who will generate the allocation sequence, who will enrol participants, and who will assign participants to interventions	Page 5 of the manuscript.
Blinding (masking)	#17a	Who will be blinded after assignment to interventions (eg, trial participants, care providers, outcome assessors, data analysts), and how	Page 9 of the manuscript.
Blinding (masking): emergency unblinding	#17b	If blinded, circumstances under which unblinding is permissible, and procedure for revealing a participant's allocated intervention during the trial	Page 5 of the manuscript.
**Methods: Data collection, management, and analysis**			
Data collection plan	#18a	Plans for assessment and collection of outcome, baseline, and other trial data, including any related processes to promote data quality (eg, duplicate measurements, training of assessors) and a description of study instruments (eg, questionnaires, laboratory tests) along with their reliability and	Page 7-8 of the manuscript.

For peer review only

		validity, if known. Reference to where data collection forms can be found, if not in the protocol	
Data collection plan: retention	#18b	Plans to promote participant retention and complete follow-up, including list of any outcome data to be collected for participants who discontinue or deviate from intervention protocols	Page 8 of the manuscript.
Data management	#19	Plans for data entry, coding, security, and storage, including any related processes to promote data quality (eg, double data entry; range checks for data values). Reference to where details of data management procedures can be found, if not in the protocol	Page 9 of the manuscript.
Statistics: outcomes	#20a	Statistical methods for analysing primary and secondary outcomes. Reference to where other details of the statistical analysis plan can be found, if not in the protocol	Page 9 of the manuscript.
Statistics: additional analyses	#20b	Methods for any additional analyses (eg, subgroup and adjusted analyses)	Page 9 of the manuscript.
Statistics: analysis population and missing data	#20c	Definition of analysis population relating to protocol non-adherence (eg, as randomised analysis), and any statistical methods to handle missing data (eg, multiple imputation)	Page 9 of the manuscript.

For peer review only

Methods: Monitoring

Data monitoring: formal committee	#21a	Composition of data monitoring committee (DMC); summary of its role and reporting structure; statement of whether it is independent from the sponsor and competing interests; and reference to where further details about its charter can be found, if not in the protocol. Alternatively, an explanation of why a DMC is not needed	Page 9 of the manuscript.
Data monitoring: interim analysis	#21b	Description of any interim analyses and stopping guidelines, including who will have access to these interim results and make the final decision to terminate the trial	Page 9 of the manuscript.
Harms	#22	Plans for collecting, assessing, reporting, and managing solicited and spontaneously reported adverse events and other unintended effects of trial interventions or trial conduct	Page 6 of the manuscript.
Auditing	#23	Frequency and procedures for auditing trial conduct, if any, and whether the process will be independent from investigators and the sponsor	Page 9-10 of the manuscript.

Ethics and dissemination

For peer review only

Research ethics approval	#24	Plans for seeking research ethics committee / institutional review board (REC / IRB) approval	Page 9-10 of the manuscript.
Protocol amendments	#25	Plans for communicating important protocol modifications (eg, changes to eligibility criteria, outcomes, analyses) to relevant parties (eg, investigators, REC / IRBs, trial participants, trial registries, journals, regulators)	Page 10 of the manuscript.
Consent or assent	#26a	Who will obtain informed consent or assent from potential trial participants or authorised surrogates, and how (see Item 32)	Page 12 of the manuscript.
Consent or assent: ancillary studies	#26b	Additional consent provisions for collection and use of participant data and biological specimens in ancillary studies, if applicable	n/a.
Confidentiality	#27	How personal information about potential and enrolled participants will be collected, shared, and maintained in order to protect confidentiality before, during, and after the trial	Page 10 of the manuscript.
Declaration of interests	#28	Financial and other competing interests for principal investigators for the overall trial and each study site	Page 12 of the manuscript.
Data access	#29	Statement of who will have access to the final trial dataset, and disclosure of contractual agreements that limit such access for investigators	Page 12 of the manuscript.

For peer review only

Ancillary and post trial care	#30	Provisions, if any, for ancillary and post-trial care, and for compensation to those who suffer harm from trial participation	n/a
Dissemination policy: trial results	#31a	Plans for investigators and sponsor to communicate trial results to participants, healthcare professionals, the public, and other relevant groups (eg, via publication, reporting in results databases, or other data sharing arrangements), including any publication restrictions	Page 10 of the manuscript.
Dissemination policy: authorship	#31b	Authorship eligibility guidelines and any intended use of professional writers	Listed on page11 in the title page.
Dissemination policy: reproducible research	#31c	Plans, if any, for granting public access to the full protocol, participant-level dataset, and statistical code	n/a
**Appendices**			
Informed consent materials	#32	Model consent form and other related documentation given to participants and authorised surrogates	n/a
Biological specimens	#33	Plans for collection, laboratory evaluation, and storage of biological specimens for genetic or molecular analysis in the current trial and for future use in ancillary studies, if applicable	n/a Biological specimens are none.

For peer review only

None The SPIRIT Explanation and Elaboration paper is distributed under the terms of the Creative Commons Attribution License CC-BY-NC. This checklist can be completed online using https://www.goodreports.org/, a tool made by the EQUATOR Network in collaboration with Penelope.ai

For peer review only
